# Evaluation of Exploratory Fluid Biomarker Results from a Phase 1 Senolytic Trial in Mild Alzheimer’s Disease

**DOI:** 10.21203/rs.3.rs-3994894/v1

**Published:** 2024-03-08

**Authors:** Valentina R. Garbarino, Juan Pablo Palavicini, Justin Melendez, Nicolas Barthelemy, Yingxin He, Tiffany F. Kautz, Marisa Lopez-Cruzan, Julia J. Mathews, Peng Xu, Bin Zhan, Afaf Saliba, Nagarjunachary Ragi, Kumar Sharma, Suzanne Craft, Ronald C. Petersen, Jair Machado Espindola-Netto, Ailing Xue, Tamara Tchkonia, James L. Kirkland, Sudha Seshadri, Arash Salardini, Nicolas Musi, Randall J. Bateman, Mitzi M. Gonzales, Miranda E. Orr

**Affiliations:** 1 Glenn Biggs Institute for Alzheimer’s & Neurodegenerative Diseases, University of Texas Health Science Center at San Antonio, San Antonio, TX, USA; 2 Department of Cell Systems and Anatomy, University of Texas Health Science Center at San Antonio, San Antonio, TX, USA; 3 Department of Medicine, University of Texas Health Science Center at San Antonio, San Antonio, TX, USA; 4 Barshop Institute for Longevity and Aging Studies, University of Texas Health San Antonio, San Antonio, TX, USA; 5 Department of Neurology, Washington University School of Medicine, St. Louis, MO, USA; 6 Tracy Family SILQ Center for Neurodegenerative Biology, St. Louis, MO, USA; 7 Department of Psychiatry, University of Texas Health Science Center at San Antonio, San Antonio, TX, USA; 8 Department of Genetics and Genomic Sciences, Icahn School of Medicine at Mount Sinai, New York, NY, USA; 9 Mount Sinai Center for Transformative Disease Modeling, Icahn School of Medicine at Mount Sinai, New York, NY, USA; 10 Center for Precision Medicine, University of Texas Health Science Center at San Antonio, San Antonio, TX, USA; 11 Department of Internal Medicine Section on Gerontology and Geriatric Medicine, Wake Forest School of Medicine, Winston-Salem, NC, USA; 12 Department of Neurology, Mayo Clinic, Rochester, MN, USA; 13 Department of Physiology and Biomedical Engineering, Mayo Clinic, Rochester, MN, USA; 14 Department of Internal Medicine, Mayo Clinic, Rochester, MN, USA; 15 Department of Neurology, University of Texas Health Science Center at San Antonio, San Antonio, TX, USA; 16 Department of Neurology, Boston University School of Medicine, Boston, MA, USA; 17 Department of Medicine, Cedars-Sinai Medical Center, Los Angeles, CA, USA; 18 Department of Neurology, Cedars Sinai Medical Center, Los Angeles, CA, USA; 19 Salisbury VA Medical Center, Salisbury, NC, 28144, USA

## Abstract

Senescent cell accumulation contributes to the progression of age-related disorders including Alzheimer’s disease (AD). Clinical trials evaluating senolytics, drugs that clear senescent cells, are underway, but lack standardized outcome measures. Our team recently published data from the first open-label trial to evaluate senolytics (dasatinib plus quercetin) in AD. After 12-weeks of intermittent treatment, we reported brain exposure to dasatinib, favorable safety and tolerability, and modest post-treatment changes in cerebrospinal fluid (CSF) inflammatory and AD biomarkers using commercially available assays. Herein, we present more comprehensive exploratory analyses of senolytic associated changes in AD relevant proteins, metabolites, lipids, and transcripts measured across blood, CSF, and urine. These analyses included mass spectrometry for precise quantification of amyloid beta (Aß) and tau in CSF; immunoassays to assess senescence associated secretory factors in plasma, CSF, and urine; mass spectrometry analysis of urinary metabolites and lipids in blood and CSF; and transcriptomic analyses relevant to chronic stress measured in peripheral blood cells. Levels of Aß and tau species remained stable. Targeted cytokine and chemokine analyses revealed treatment-associated increases in inflammatory plasma fractalkine and MMP-7 and CSF IL-6. Urinary metabolites remained unchanged. Modest treatment-associated lipid profile changes suggestive of decreased inflammation were observed both peripherally and centrally. Blood transcriptomic analysis indicated downregulation of inflammatory genes including *FOS, FOSB, IL1β, IL8, JUN, JUNB, PTGS2*. These data provide a foundation for developing standardized outcome measures across senolytic studies and indicate distinct biofluid-specific signatures that will require validation in future studies.

ClinicalTrials.gov: NCT04063124.

## Introduction

Senescent cell accumulation and the resultant pro-inflammatory senescence associated secretory phenotype (SASP) have been linked with the amyloid ß (Aß) and tau pathologies of Alzheimer’s disease and related dementias (AD; ADRDs)^1–3^. In light of this, senescent cell clearance is being explored as a novel therapeutic mechanism for AD (for review:^4,5^; ongoing trials: SToMP-AD: NCT04685590, ALSENLITE: NCT04785300; STAMINA: NCT05422885). Targeted removal of senescent cells with the senolytic therapy that has been most comprehensively characterized and studied, combined dasatinib plus quercetin (D+Q), has demonstrated successful reduction of AD-related neuropathological burden of tau-containing neurofibrillary tangles (NFTs)^1^ and Aß plaques^3^, and prevented age-associated cognitive deficits in animal models^6,7^. We previously reported the outcomes of a 12-week open-label pilot study (Senolytic Therapy to Modulate the Progression of AD: SToMP-AD; NCT04063124), aimed at determining blood-brain barrier penetrance and safety and tolerability of D+Q in five study participants with early-stage symptomatic AD^8^. D but not Q, was detectable in the cerebrospinal fluid (CSF), and the intervention was well-tolerated^8^. Additionally, we reported data on secondary outcomes relevant to AD biomarkers, the SASP, cognitive function, and brain imaging^8^. As a follow-up to our original publication, we performed exploratory proteomic, lipidomic, and transcriptomic analyses on blood, CSF, and urine samples collected at baseline and post-treatment. The results provide a more comprehensive understanding of the systemic and central nervous system (CNS) effects of senolytic therapy in AD.

The heterogeneous, cell-type, and context specific phenotype of senescent cells presents a barrier in identifying appropriate biomarkers to monitor target engagement in clinical trials^9^. With this in mind, we aimed to utilize samples from the SToMP-AD pilot study to identify biomarkers that may be modulated by senolytic therapy and can be further validated by future trials on senolytics in AD and other CNS conditions. Herein, we present the baseline versus post-treatment outcomes from mass spectrometry analysis of a comprehensive list of Aß and phosphorylated tau proteins in CSF; quantitative assays for measuring levels of cytokines and chemokines linked to SASP or other hallmarks of aging measured in plasma, CSF, and urine; metabolite analysis in urine; and mass spectrometry analysis of lipidomic changes in plasma and CSF. Given that chronic stress is a known driver of both cellular senescence^10^ and the pathologies and symptomology of AD^11,12^, we also measured transcriptomic changes in a chronic stress-related gene profile termed the conserved transcriptional response to adversity (CTRA) in peripheral blood mononuclear cells (PBMCs). This was done to probe the therapeutic utility of senolytics to address the chronic-stress drivers of AD.

## Results

### Study Participant Sample Availability:

Five individuals, aged 70–82 years old, with a clinical diagnosis of early-stage dementia due to AD were enrolled in the SToMP-AD pilot study. Baseline and post-treatment samples were available from all five participants for tau phosphorylation protein measures (in CSF), and lipidomics measures (plasma and CSF), while four of the five participant samples were available for Aß isoform analysis, urine metabolites, and CTRA transcriptomic analysis. Color coding of samples matches those that are described in Table 2 of the parent publication^8^, which provides additional information on participant characteristics.

### Aß and tau Biomarker Measures in CSF:

Baseline and post-treatment Aß42 and Aß40 levels in CSF were measured by mass spectrometry and the Aß42:40 ratio was calculated for each participant as a surrogate measure of amyloid deposition (**Supplementary Figure 1a-d**). No statistically significant changes were observed (*P* > 0.05). Similarly, baseline and post-treatment CSF levels of differentially phosphorylated tau and corresponding endogenous peptides were measured (pT153, tau 151–155, pT181, tau 181–190, pS199, pS202, pT205, pS208, tau 195–210, pT217, tau 212–221, pT231, tau 226–230). The phosphorylated tau occupancy at different tau residues (pT111/T111, pT153/T153, pT181/T181, pS199/S199, pT205/T205, pS208/S208, pT217/T217, pT231/T231), as well as the levels of microtubule binding region (MTBR) for tau 212–221 (MTBR-tau212–221) and tau 243–254 (MTBR-tau243–254, an indicator of tau tangles^13^) displayed no statistically significant changes across time points (**Supplementary Figure 2 a-j** and **Supplementary Table 1**).

### Baseline ADRD Biomarkers Associated with Cerebrospinal Fluid Concentrations of Dasatinib:

**Figure 1.**
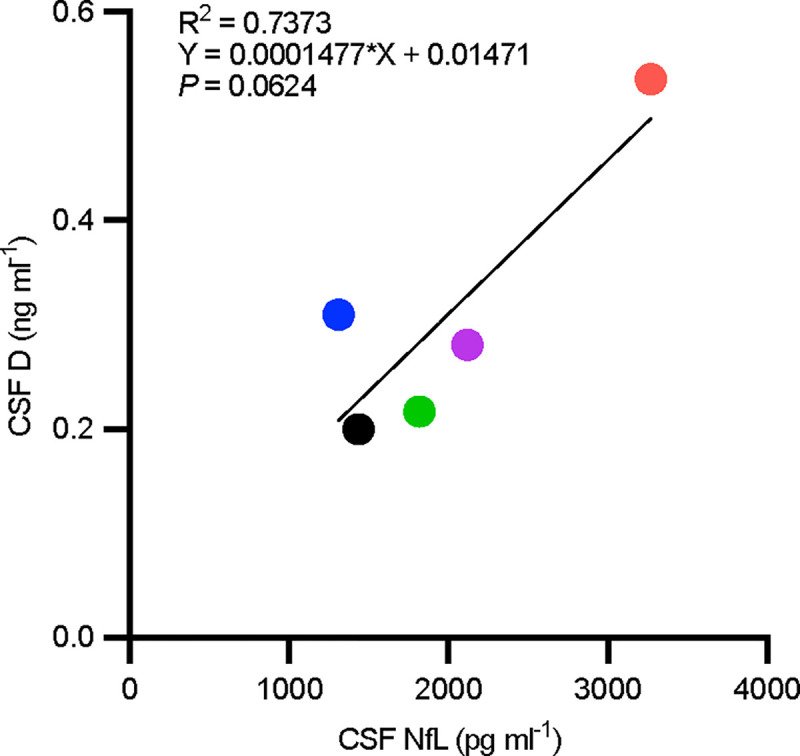
Correlation plot of cerebrospinal fluid (CSF) dasatinib (D) *versus* neurofilament light chain (NfL) levels. Post-treatment dasatinib (D) level correlation with baseline cerebrospinal fluid neurofilament light chain (NfL) derived from simple linear regression. R^2^ = 0.7373; *P* = 0.0624.

We observed a non-significant trend for increased levels of D into the CSF by individuals with higher baseline CSF NfL concentrations, a marker of neurodegeneration^14^ (R^2^ = 0.7373; *P* = 0.0624; Figure 1). There was no correlation between CSF levels of D and measures of Aß or tau as indicated by *P* > 0.05 for all analytes assessed.

### Senescence Associated Secretory Factors in Plasma, CSF, and Urine:

**Figure 2.**
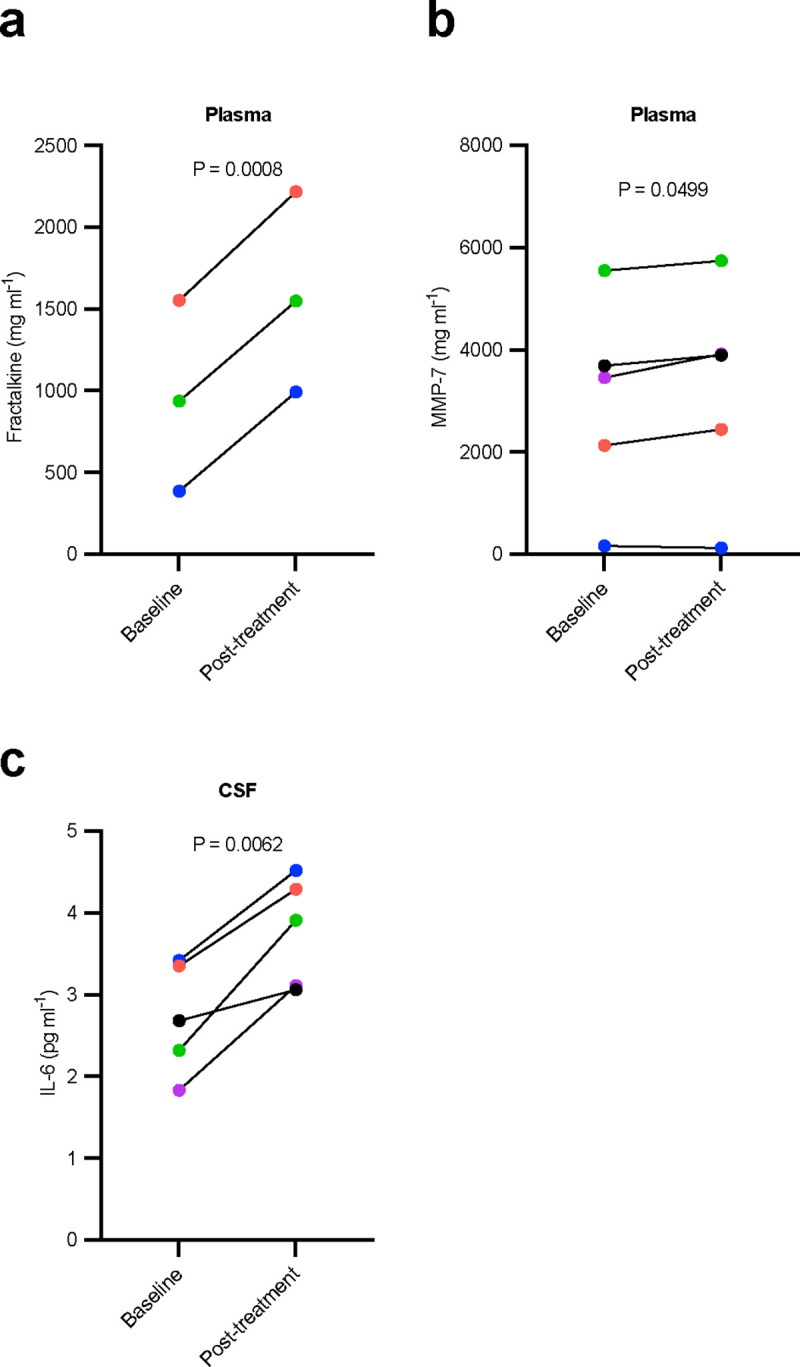
Inflammatory protein levels altered by dasatinib plus quercetin (D+Q) treatment measured by Luminex^®^ protein platform. **a-d**, Effect of dasatinib plus quercetin (D+Q) on plasma and cerebrospinal fluid (CSF) inflammatory markers. Mean difference (95% CI): **a**, plasma fractalkine, 629 (549.90 to 705.60); **b**, plasma MMP-7, 226 (0.198 to 452.90); **c**, CSF IL-6, 1.06 (0.500 to 1.616). Baseline to post-treatment changes were assessed using two-sided paired sample t-tests, P<0.05, N = 3–5, color coded by participant. Mean difference = post-treatment - baseline; 95% CI, for the post *versus* baseline mean difference. No correction for multiple comparisons was made due to small sample size.

Baseline to post-treatment paired samples t-tests revealed statistically significant increases in four proteins analyzed from plasma, CSF and urine samples by multiplex protein analysis. Though these outcomes would not have survived corrections for multiple comparisons, there were significant post-treatment increases in the following multifunctional biofluid proteins: plasma fractalkine and MMP-7, and CSF IL-6; other analytes that displayed trends toward change at *P <* 0.1 were plasma eotaxin and VEGF (Figure 2 and **Supplementary Table 2**).

### Metabolic Analysis in Urine Samples:

Thirteen of the seventeen urinary amino acid and related metabolites measured via mass spectrometry were detectable in baseline and posttreatment samples from 4 of the 5 study participants, as a paired sample was not available at baseline for one of the participants. Baseline to post-treatment paired samples t-tests showed no statistically significant differences in any of the metabolites across time points (**Supplementary Table 3)**. Sulpiride, glutamine, glutamic acid, and nicotinic acid were excluded from analyses as urinary concentration of these metabolites was below the limit of quantitation (0.1 μM).

### Lipidomics Analysis in Plasma and CSF:

**Figure 3:**
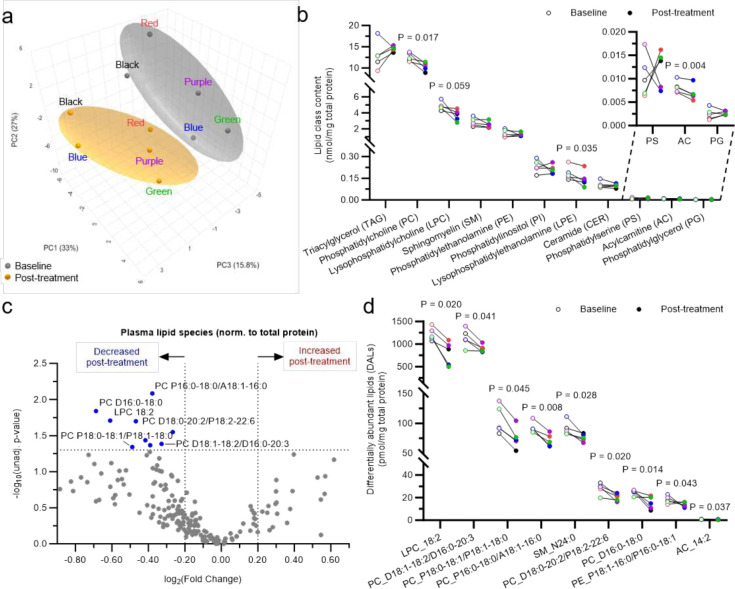
Effects of dasatinib plus quercetin (D+Q) treatment on the circulating plasma lipidome normalized to total protein concentration. **a-d**, Effects of dasatinib plus quercetin (D+Q) treatment on the circulating plasma lipidome normalized to total protein content. Plasma lipidome was assessed using multidimensional mass spectrometry-based shotgun lipidomics. **a,** MetaboAnalyst unsupervised PCA plot reducing all plasma lipid species data into three dimensions. Baseline and post-treatment groups are color-coded in gray and orange respectively, subjects are color coded to match color code assignments across all figures. **b,** All 11 lipid classes assessed in plasma samples. Paired samples are connected with a line. **c**, Volcano plot comparing all 194 plasma lipid species at baseline and post-treatment. **d**, Plot of the nine differentially abundant lipids (DALs) lipid species significantly decreased from baseline to post-treatment. Paired samples are connected with a line, each color represents a different subject (N=5). Only P < 0.1 are shown.

MetaboAnalyst unsupervised metadata analysis on plasma lipidomics data using all 194 detected lipid species revealed that among all factors assessed (pre/post senolytic treatment, biological sex, subject, age, and pre/post MoCA scores), biological sex had the strongest impact on the circulating lipidome, followed by senolytic treatment (**Supplementary Figure 3a**). Total protein content in both plasma and CSF was stable across timepoints (**Supplementary Figure 3b**). Previous metadata analysis had revealed that sex separation was largely driven by principal component 1 (PC1: 33%) (**Supplementary Figure 3c**). Subsequent analyses were performed following MetaboAnalyst paired one factor module using transformed and scaled lipid mass levels expressed relative to plasma total protein content for both time points (**Supplementary Figure 3c**). Principal component analysis (PCA) 3D scatter-plotting revealed an evident separation between baseline and post-treatment sample clusters, indicating that senolytic treatment had a notable impact on the circulating lipidome as a whole (Figure 3a). This separation was primarily driven by PC2 and PC3 (27% and 15.8%, respectively) (Figure 3a).

Paired comparisons between baseline and post-treatment plasma samples at the lipid class level revealed that out of the 11 lipid classes analyzed, three classes were significantly altered post-treatment when applying an unadjusted *P* < 0.05 cut-off (Figure 3b). These included phosphatidylcholine (PC), the most abundant phospholipid in circulation and major constituent of lipoprotein membranes, which decreased post-treatment by 17% (*P*= 0.017), a biologically relevant amount considering that circulating PC levels are tightly regulated; lysophosphatidylethanolamine (LPE), a cleavage product of the second most abundant phospholipid (PE), which decreased by 22% (*P* = 0.035); and acylcarnitine an intermediate of fatty acid oxidation present at very low levels in circulation that was decreased by 16% (*P* = 0.004). The low acylcarnitine levels in circulation is consistent with previous reports and with the fact that in plasma there are very few cell-free mitochondria or peroxisomes, the sites where acylcarnitines are produced and reside. Finally, lysophosphatidylcholine (LPC), the most abundant lysolipid in the circulation associated with inflammation, apoptosis, oxidative stress, and atherosclerosis^15–19^, displayed a 24% decreasing trend (*P* = 0.059) (Figure 3b).

Paired comparisons between baseline and post-treatment plasma samples at the lipid species level revealed nine differentially abundant lipid species (DALs) when applying an unadjusted *P* < 0.05 cut-off, all decreased post-treatment (Figure 3c). More than half of these DALs were PC species of high, medium, or low abundance, including both diacyl and plasmalogen species (Figure 3d). Additional DALs included the second most abundant LPC species (18:2), which was significantly reduced by 35%, and the fourth most abundant acylcarnitine species (14:2) (Figure 3d).

**Figure 4.**
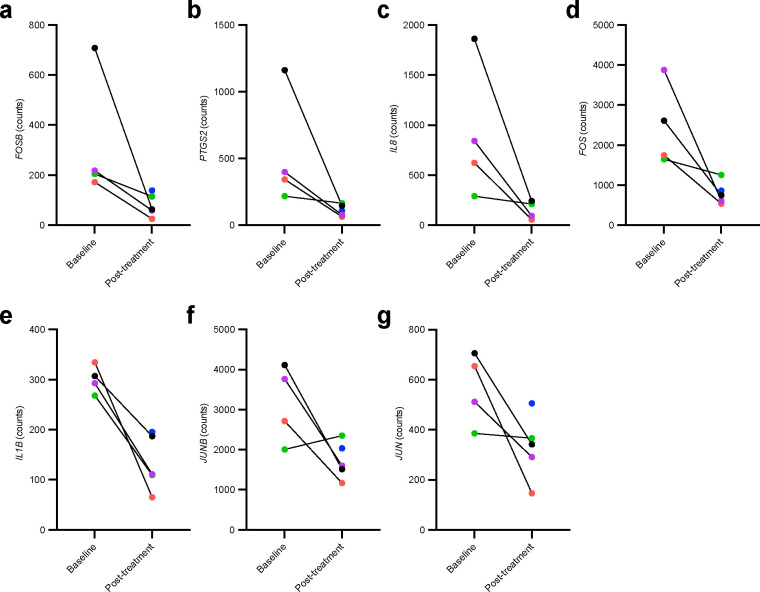
Baseline and post-treatment significantly differentially expressed Conserved Transcriptional Response to Adversity (CTRA) gene counts in peripheral blood mononuclear cell samples measured with nanoString nCounter XT CodeSet custom CTRA gene expression panel. **a-g**, Effects of dasatinib plus quercetin (D+Q) on the expression of Conserved Transcriptional Response to Adversity (CTRA) gene counts measured in peripheral blood mononuclear cell (PBMC) samples. Seven inflammatory genes were significantly decreased post-treatment. Mean difference (B-statistic): **a**, *FOSB*, −218.87 (−0.713); **b**, *PTGS2*, −377.76 (−1.177); **c**, *IL-8*, −675.93 (−1.215) (**d**) *FOS*, −1579.32 (−1.669); (**e**) *IL-1B*, −152.94 (−1.922), (**f**) *JUNB*, −1267.29 (−3.546) (**g**) *JUN*, −505.57 (−3.754). Baseline to post-treatment changes were assessed using two-sided paired sample t-tests, *P* < 0.05, N = 4, color coded by participant. Paired baseline and post-treatment measures existed for all but one of the participants (blue) for whom only a post-treatment sample was collected. Mean difference = post-treatment - baseline. No correction for multiple comparisons was made due to small sample size.

It is important to note that if samples are normalized to plasma volume, no separation is observed by PCA (**Supplementary Figure 4a**), presumably due to the higher intrinsic variability/noise associated with normalizing analytic results to sample volume. It is also worth mentioning that when normalized to plasma volume, only one class was significantly altered by treatment: triacylglyceride (TAG), which was increased post-treatment by 23% (**Supplementary Figure 4b**; *P* = 0.022). These results are consistent with those obtained via lipid panel lab testing, which are also expressed by volume, where a 28% post-treatment increasing trend was observed (*P* = 0.064) as previously reported^8^. Additional analysis at the lipid subclass level revealed that long-chain fatty acyl-containing TAGs were significantly increased (**Supplementary Figure 4c)**. Moreover, consistent with the above-described protein content-based results, LPC also tended to decrease when normalizing to volume (**Supplementary Figure 4b**; *P* = 0.066). Lipid subclass analyses revealed that long-chain fatty acyl-containing LPCs tended to decrease (**Supplementary Figure 4d**). Finally, the vast majority of the volume-normalized DALs (4 out of 5) were TAG species, which increased post-treatment (Figure 4e–f).

Lastly, unsupervised dimensionality reduction (PCA) of CSF lipidomics data using all 79 detected lipid species normalized to total protein content revealed no separation between baseline and post-treatment samples (**Supplementary Figure 5a**), implying a lack of global senolytic effect at the whole CSF lipidome level. At the lipid class level, paired comparisons revealed that none of the nine lipid classes assessed in the CSF were significantly altered post-treatment. At the lipid species level, paired comparisons revealed five DALs when applying an unadjusted *P* < 0.05 cut-off, including the second most abundant LPC species (16:1) in the CSF that was reduced by 43% post-treatment (**Supplementary Figure 5c-d**; *P* = 0.014), the largest effect observed by magnitude. Notably, this same LPC species came up as the most important feature on a partial least squares-discriminant analysis (PLS-DA), a supervised dimensionality reduction method that was able to largely separate baseline and post-treatment samples (**Supplementary Figure 5b**). The other four DALs that were significantly increased post-treatment included two PC species that were increased by 16% (D16:0–16:0, the second most abundant PC species in CSF) and 21% (D16:1–16:0/D14:1–18:0, a medium abundant PC species) post-treatment (**Supplementary Figure 5c-d**). When CSF lipidomics data were normalized to volume content, none of lipid classes nor species were significantly altered. Only one species (LPC 16:1) tended to change post-treatment (41% decrease, *P* = 0.080). The decrease of LPC 16:1 in the CSF, which reached significance when normalized to protein content as mentioned above, was reminiscent and consistent with the decreases observed on other lysophospholipid species in circulation.

### Effects of Senolytic Therapy on a Transcriptomic Stress Profile:

Transcriptomic analysis of the PBMC samples revealed baseline to post-treatment downregulation of seven of the 19 inflammatory related genes included in the Conserved Transcriptional Response to Adversity (CTRA) transcriptomic stress profile; *FOSB*, *PTGS2*, *IL8*, *FOS*, *IL1β*, *JUNB*, and *JUN* (*P* < 0.05; Figure 4, **Supplementary Table 4**). No significant differences were seen between time points for genes within the Type I interferon or antibody synthesis categories, though *IFI27L1, IFITM1,* and *IFITM4P* showed trends toward an increase (*P =* 0.058; *P =* 0.110; *P =* 0.110, respectively) (**Supplementary Table 4**).

## Discussion

In the last few years, senolytics have been translated from rodent studies to early-stage clinical trials^20^. Given the recent emergence of this therapeutic strategy, the methodology to reliably identify senescent cell presence, clearance, and the related clinical efficacy is still under development. Here we used biofluids from the first in human senolytic pilot for AD to quantify various types of analytes across multiple accessible biofluids (plasma, CSF, and urine) with the goal of determining which may be most useful and informative for exploration in future trials. Overall, no differences were detected between baseline and post-treatment in assessments for ADRD Aß and tau biomarkers, but we observed a potentially interesting trend between baseline NfL protein levels, a marker of neurodegeneration^21^, and post-treatment D levels in the CSF. We also observed biofluid-specific changes in treatment response with blood analytes showing the greatest promise. Of particular interest were observed significant and/or strong trends for several blood SASP factors, lipids, and CTRA measures. These data will help direct senescence biomarker/gerodiagnostic development and refinement that can be used as a guide for outcome measures to be included in future trials.

To obtain a comprehensive analysis of ADRD biomarkers within our sample population before and after senolytic therapy, we analyzed levels of Aß and phosphorylated tau protein species and fragments currently most predictive of amyloid plaques and NFT pathology^22–26^. CSF levels of Aß and phosphorylated tau, as assessed by immunoassays, correlate with AD disease state and neurodegenerative pathology, but more disease specific information can be gleaned from assessment of the specific post-translational modifications of tau^27,28^. The Aß and tau biomarkers in our small open-label trial measured by mass spectrometry were unchanged from baseline to post-treatment. These results are consistent with those presented in initial SToMP-AD pilot trial publication^8^, which were measured with the Simoa HD-X analyzer (Quanterix, Lexington, MA) and Fujirebio G1200 (Malvern, PA, lumipulse assay). Given the high precision and accuracy of these mass spectrometry assays, we interpret that the senolytic treatment did not change CSF amyloid ß, multiple phospho-tau species, or MTBR-243 tau, a measure that robustly correlates with tau tangles.

Throughout the disease course, Aß^29^ and tau^30^ biomarkers gradually change over many years to decades, but levels (particularly of Aß) are dynamic, with previous studies demonstrating that alterations in production and clearance are observable in response to a number of interventions even within a short period of time (< 4 weeks)^31,32^. Unless plaque and tangle pathologies are changing rapidly, significant alterations in these biomarkers may not be expected in the 12-week intermittent treatment period of the pilot study. We note that it is encouraging that AD biomarkers did not worsen, but remained stable, across the study duration. These data indicate the intervention did not exacerbate disease, and may have a slowing effect of disease progression as seen in mouse studies^1^. A longer duration study with a placebo arm is underway and will help inform disease-modifying effects of senolytic therapy^33^.

With advancing neurodegenerative disease, blood-brain barrier integrity becomes compromised^34^. Elevations of NfL in serum have been linked with loss of blood-brain barrier integrity in multiple sclerosis^35^, but the degree to which CSF NfL is predictive of blood-brain barrier integrity in AD is unclear^36,37^. The observed trend for increased levels of D in CSF in participants with higher levels baseline CSF NfL highlights how factors relevant to AD severity and neurodegenerative disease progression should be considered in regard to therapeutic effect and efficacy within and across individuals. Understanding the implications of blood-brain barrier integrity on the potential penetrance, uptake, and metabolism of senolytic compounds is a critical pharmacological factor that will require further study in both basic science and clinical research settings to ensure safety and efficacy in an early AD study population. Future studies with a larger sample size will be necessary to determine if there is a true correlation between the concentration of NfL or other ADRD biomarkers with the concentration of D in the central nervous system. Well-designed pharmacokinetic/pharmacodynamic studies will be necessary to fully understand the distribution and metabolism of senolytic compounds in healthy controls versus those with neurodegenerative disease. Additionally, the inclusion of measures more directly informative about blood-brain barrier integrity could be considered in future trials, including dynamic contrast enhanced MRI (DCA-MRI) imaging analysis^38,39^ or CSF biomarkers such as PDGFRß^40^.

In our first publication reporting the results of the phase 1 senolytic trial in an early AD population, we presented the effects of D+Q senolytic therapy on plasma and CSF SASP factors measured by Quanterix and Lumipulse^8^. To futher investigate how senolytics affect SASP factors and resolve discordance in the literature concerning result consistency across various assay platforms, we employed multiplex magnetic bead immunoassays to measure a wider array of cytokines, chemokines, growth factors, and proteinases in plasma, CSF, and urine samples. In agreement with our initial report, the majority of protein biofluid markers remained unchanged baseline to post-treatment, but we did observe three proteins which were significantly elevated post-treatment (plasma MMP-7 and fractalkine; and CSF IL-6). Though elevation of cytokines is generally indicative of inflammation, these proteins play critical roles in necessary and beneficial immunological responses in neurodegenerative disease and senescent cell clearance. For example, upregulation of fractalkine, a chemokine that dampens the pro-inflammatory state of microglia and plays a role in adult neurogenesis, has been shown to reduce tau pathology and neurodegeneration in an animal model^41^, and elevated plasma fractalkine levels were protective in a stroke population^42^. Elevated plasma IL-6 is indicative of increased inflammation and associated with the progression and pathologies of Alzheimer’s disease^43^, but short-term elevation may be indicative of positive biological effects in regard to the mechanisms of action of senolytics and target engagement. A previous trial which utilized D+Q in a population with diabetic kidney disease reported reduced levels of plasma IL-6 after only 3 days of senolytic treatment^44^. However, these measures were made from samples that were collected 11 days after the final dose of medication, whereas in our study we report levels from samples collected immediately after the final dose of D+Q, meaning participants had started their final drug administration cycle 24 hours prior to biofluid sample collection. It is reasonable to speculate that the increase in these inflammatory related markers may be indicative of acute inflammatory response induced by D+Q senescent cell ablation or “senolysis”. Further, a few additional SASP related plasma proteins revealed nonsignificant modest decreases post D+Q treatment (*e.g*., eotaxin, MCP-1, VEGF), which have been shown previously to elevated in AD^45^, and negatively associated with memory in MCI and AD^46^. It will be important for future trials to measure these SASP related factors at multiple time points after completing D+Q to distinguish the acute versus chronic effects of treatment^33^ (ClinicalTrials.gov: NCT04685590). Within these samples, we previously reported higher pre- to post-treatment CSF IL-6 levels as assessed by the Mesoscale Discovery U-Plex Biomarker Group 1 (hu) 71-plex panel. Our data presented here indicate that IL-6 did not change in plasma or urine to highlight the importance of biofluid consistency when measuring markers of SASP as different cytokines changes were observed across plasma, CSF, and urine. Though interpretation of these data is made difficult by the small sample size and lack of a control group for comparison, these data nevertheless provide further evidence to guide future experimental design and methods.

Urinary metabolite profiles have been linked with AD and proposed as potential biomarkers for mild cognitive impairment and AD^47^. Recent studies from our group indicate the utility of urine metabolomics to understand complex diseases^48^. In our study, urinary metabolomics were unchanged. Although larger placebo-controlled studies are necessary, the preliminary results are encouraging as no changes in urinary metabolites associated with adverse events or AD pathogenesis were observed.

Due to the high lipid content of the brain and the critical role that lipids play in the integrity and function of cell membranes, lipidomic measurements offer important insights to brain health and disease processes^49^. Recent work indicates that lipid metabolism and homeostasis becomes dysregulated with advancing neurodegeneration^50,51^ and general lipidomic dysregulation in AD^52,53^, which highlights the potential utility of lipid measurements to be used as AD biomarkers. Further, lipidomic dysregulation has been posed as a driver of cellular senescence and associated inflammation^54,55^, which make understanding the lipidome in AD important from the perspective of both biomarker potential and AD driving insult to target therapeutically. Lipid expression relative to total protein content is the most commonly used and preferred normalization method for lipidomics studies^56–58^, particularly for the assessment of lipids in plasma, where virtually all lipids are bound to protein transporters (*e.g*., albumin and lipoproteins), but in clinical settings lipid levels are often normalized to sample volumes. Therefore, we conducted the analyses both ways to determine the most appropriate method to detect lipidomic changes in response to senolytic therapy. Despite the relatively short duration of senolytic treatment in this study, unsupervised lipidomics analysis revealed global post-treatment effects on the circulating lipidome when expressed relative to protein content that were of potential biological relevance. Although senolytic treatment altered the circulating lipidome, the effect was relatively mild as it was no longer observed when more stringent statistical methods were applied, which may reflect the relatively short duration of the study intervention. When lipid levels were normalized to total protein, senolytic treatment led to significant decreases in total LPE content and a major LPC species in plasma. The reduction in circulating lysophospholipids observed following D+Q treatment is suggestive of reduced inflammatory pathways and consistent with the obtained CTRA transcriptomic results. These results are consistent with potential positive effects of senolytics on a broad range of biological aging measures. Additionally, we found an increase in circulating TAG when lipid concentrations were expressed relative to plasma volume. The fact that long-chain, but not very long-chain containing TAG species, were altered, suggests that increased *de novo* lipid synthesis may be responsible for the observed increase in TAG. In the SToMP-AD pilot study, we previously reported a trend towards higher post-treatment total triglyceride levels in the clinical lipid panel (*P* =0.064; Supplementary Table 1 of the parent trial results^8^), providing complimentary evidence for plasma lipid changes. We also observed decreases per protein content of total PC, the most abundant phospholipid on lipoprotein particle membranes. Taken together, TAG and PC data suggest that senolytic therapy may illicit biological modifications of lipid lipoprotein profiles. Finally, the observed decreases in circulating acylcarnitines point toward a putative effect of senolytics on energy metabolism, specifically on fatty acid oxidation. These potential changes require further validation to determine if lipidomic outcomes may be utilized as sensitive biomarker indicators for senescent cell clearance in future trials.

Even through the short senolytic treatment did not have global effects on the CSF lipidome, it led to robust decreases in a specific lysophospholipid species (LPC 16:1). These results may be biologically relevant given the high abundance of this LPC species that is consistent with the decreases in lysophospholipids observed in circulation and the expected anti-inflammatory effects of senolytics. Taken together, our results place lipids as particularly sensitive and clinically valuable markers, as consistent with preclinical studies^59,60^ (for review: ^61,62^).

Senolytics have been proposed as a potential therapeutic for chronic-stress induced memory deficits^10^. The CTRA represents a transcriptomic profile activated by chronic stress that is measured in circulating PBMCs^63^. Specifically, the CTRA transcriptomic pattern is defined by relative upregulation of 19 inflammatory genes, and relative downregulation of 31 type-1 interferon response genes and three antibody synthesis genes^63,64^ as displayed in **Supplementary Table 4**. The CTRA has been proposed as a potential predictive biomarker for disease risk and pathogenesis related to health conditions impacted by inflammatory and interferon response alterations including cancer and heart disease^65^, and may have utility as an indicator of AD progression^64^. We were interested in assessing the utility of the CTRA as an outcome measure relevant to senescence and senolytic response in AD. Our study identified a baseline to post-treatment reduction in *FOSB, PTGS2, IL8, FOS, IL1β, JUNB*, and *JUN* expression in PBMCs. Elevated levels of each of these transcripts have been associated with senescence and SASP secretion^66–72^, with our observed decreases suggesting downregulation of pathways involved in inflammation and cell fate decisions^73,74^ and provide exciting evidence for senolytic target engagement, at least peripherally in this small pilot study. The consistent decrease in expression across all seven of these differentially expressed inflammatory markers is encouraging from a therapeutic standpoint as chronic peripheral inflammation^75,76^, and even psychological stress^77^, is associated with AD. Our preclinical study of senolytics for AD previously reported an upregulation in *IL1β* in the brain associated with NFTs that decreased with D+Q^1^. An independent preclinical study also reported downregulation of *IL1β* in the brain in response to D+Q^3^. Recent publications indicate that treatments with D+Q in animal models reduce markers of inflammation associated with the pro-inflammatory senescence associated secretory proteins, including IL1β (as measured in intestinal and adipose tissue in mouse models)^78,79^, which was also reduced in our hands.

We also observed non-significant increases in *IFI27L1, IFITM1*, and *IFITM4P* type-1 interferon response genes. Given that these genes are typically down-regulated in CTRA, the data provide additional support that senolytics may be positively impacting this chronic stress pathway. While the gene expression findings in our study suggest that senolytics may impact the CTRA, significant changes would not have survived multiple comparisons correction; our preliminary findings require further replication in studies designed to assess this endpoint. Additional work to better understand the CTRA transcriptomic profile in AD, in general, will be necessary to understand the utility of this panel as a biomarker and to more fully understand the implications of changes in these markers in response to senolytic therapy. Our team is currently working on establishing these baseline measures in AD, and will assess the senolytic-associated change in the larger Phase 2 SToMP-AD trial (NCT04685590)^33^. Notably, other trials of particular interest in validating changes in CTRA are those focused on D+Q in treatment resistant depression (NCT05838560)^80^.

In summary, the stable baseline and post-treatment Aß and tau species measured with mass spectrometry provide evidence that senolytic therapy does not exacerbate AD. These findings also underscore the importance of identifying biomarkers specific to senolytic treatment as AD surrogate measures are predicted to be downstream of senescent cell clearance. The additional experimental measures comparing the baseline versus post-senolytic outcomes from this study suggest that senolytics, D+Q, may, at least acutely, increase markers of inflammation, while reducing circulating inflammatory lipid species and transcriptomic markers of the CTRA. These supplementary measures provide clues that will contribute to the development of biomarker panels associated with global senescent cell clearance specifically in an AD population. Pathways and mechanisms by which D+Q may elicit a biologically relevant effect in persons with AD will likely be better distinguished from the natural AD course by including a placebo control group and longer study duration. There is immediate opportunity to confirm these biomarker findings in two on-going Phase I D+Q trials in AD (ALSENLITE: NCT04785300; STAMINA: NCT05422885) and one for treatment-resistant depression (NCT05838560). Using these exploratory outcomes as biomarkers in the ongoing fully powered, double-blind, placebo-controlled phase 2 study (SToMP-AD: NCT04685590) will better inform senolytic target engagement and therapeutic efficacy, that will guide the development and study design of future senolytic studies.

## Methods

### Study Design:

The full study protocol^81^, as well as the detailed results from the initial reporting of results of the SToMP-AD trial, have been separately published^8^. In brief summary, five individuals with early-stage AD were recruited to participate in an open-label trial which provided dasatinib (100 mg, Spruce, Bristol Meyers Squibb) and quercetin (1000 mg, Thorne Research) orally on an intermittent dosing schedule for three months. The trial was conducted in compliance with all relevant ethical regulations and the Guideline for Good Clinical Practice. D+Q were administered under Investigation New Drug (IND) 143945–0006 (to N.M.). The study protocol was approved by the UT Health San Antonio Institutional Review Board (IRB). All participants provided written informed consent with an appropriate legally authorized representative.

### Biospecimen Collection and Storage:

All plasma, CSF, and urine biospecimens utilized for these analyses were collected under fasting conditions as described previously, at baseline (Visit 1) and post-treatment (Visit 9), the morning of the second day of the final drug administration cycle^8^. Peripheral blood mononuclear cell (PBMC) isolation was performed in BD Vacutainer CPT Mononuclear Cell Preparation (CPT) Sodium Heparin tubes (Franklin Lakes, NJ) according to the manufacturer’s protocol. The resulting PBMCs were stored in three, 1 ml aliquots containing heat inactivated fetal bovine serum (Corning, NY) with 10% dimethyl sulfoxide (Corning, NY). The PBMCs were stored overnight at −80°C in a Mr. Frosty container (Nalgene, Rochester, NY) before final storage in a liquid nitrogen freezer.

### Mass Spectrometry for Amyloid and Tau Cerebrospinal Fluid Biomarkers:

Previously published methods were utilized to measure CSF Aß^82^, tau and ptau peptides, residues^22^, and HJ32.11-MTBR-tau microtubule binding regions^24^.

### Inferring Blood-Brain-Barrier Integrity with Drug and Biomarker Correlation:

Levels of CSF NfL and Dasatinib penetrance into the CSF were assayed as described in Gonzales *et al*., 2023. The Pearson r correlation between baseline NfL levels and post-treatment D were assessed for each participant by simple linear regression.

### Senescence Associated Secretory Factors in Plasma, Cerebrospinal Fluid, and Urine:

Baseline *versus* post-treatment levels of plasma, CSF, and urine biomarkers associated with SASP were evaluated at the Facility for Geroscience Analysis (FGA) at Mayo Clinic. This laboratory is part of the NIH-funded Translational Geroscience Network. Duplicate samples were analyzed using either the FLEXMAP3D Machine (Luminex) or the Ella Automated Immunoassays (Protein Simple, Bio-Techne) platforms with commercially available immunoassay kits (R&D Systems, Bio-Techne). Based on the abundance of the targeted factors, bead region, and antibody compatibility, the targets were organized into 18, 10, 6, and 5 plex plates for plasma and 15, 13, and 5 plex plates for urine. Proteins with very low abundance were measured using the ELLA Automated Immunoassay (Protein Simple/Bio-Techne) with cartridges purchased from Protein Simple/Bio-Techne. All assays were conducted according to the manufacturer’s instructions. Adiponectin was excluded from the 15-Plex urine panel due to compatibility issues with the assay beads. Urine protein levels were normalized to creatinine levels for each participant at each timepoint using commercially available kits (R&D Systems/Bio-Techne). The baseline and post-treatment levels were compared using paired-sample t-tests to assess the effect of the senolytic treatment, without correction for multiple comparisons. One participant was unable to provide a baseline urine sample, so the associated post-treatment time point was excluded from the analyses. Values below the assay’s detection limit, resulting in the absence of a matching paired sample, were excluded from the paired t-test analyses

### Metabolite Analysis in Urine Samples:

A panel of 17 urinary metabolites were measured with urine samples collected at baseline and post-treatment from 4 of the 5 study participants. Mass spectrometry (MS) protocols were slightly modified from^83,84^. Briefly, for LC/MS/MS we utilized a Thermo Q Exactive HF-X Orbitrap mass spectrometer with a Thermo Vanquish HPLC system, auto-injecting a 5 μL urine sample. For chromatography, we used an Agilent ZORBAX HILIC PLUS column with a mobile phase of components A (10 mM ammonium bicarbonate, 0.05% formic acid in Millipore water, pH=4.2) and B (0.1% formic acid in acetonitrile), with flow rate flow rate of 0.3 mL/min. The gradient ran for 12 minutes. MS settings included a 4300 V spray voltage, nitrogen gas, ion transfer tubes, and auxiliary heater at 320°C and 30°C, respectively. PRM mode was positive polarity. Data were processed using Xcalibur Quant Browser, comparing peak areas to internal standards (A/IS ratio) and a standard curve (0.01–100 μM) for concentration determination.

### Multidimensional Mass Spectrometry-Based Shotgun Lipidomics in Plasma and Cerebrospinal Fluid:

Total protein concentrations for plasma and CSF samples were determined using bicinchoninic acid (BCA) protein assay (Thermo Fisher Scientific). Lipids were extracted by a modified procedure of Bligh and Dyer extraction in the presence of internal standards, which were added based on plasma or CSF volume for each sample as previously described^58^. Lipid analyses was expressed and analyzed as per total protein content and sample volume. Lipids were assessed using a triple-quadrupole mass spectrometer (Thermo Scientific TSQ Altis) and a Quadrupole-Orbitrap^™^ mass spectrometer (Thermo Q Exactive^™^) equipped with a Nanomate device (Advion Bioscience Ltd., NY, USA) as previously described^85,86^. Briefly, diluted lipid extracts were directly infused into the electrospray ionization source through a Nanomate device, signals were averaged over a 1-min period in the profile mode for each full scan MS spectrum. For tandem MS, collision gas pressure was set at 1.0 mTorr, but the collision energy varied with the classes of lipids. Similarly, a 2- to 5-min period of signal averaging in the profile mode was employed for each tandem MS mass spectrum. All full and tandem MS mass spectra were automatically acquired using a customized sequence subroutine operated through Xcalibur software. Data processing including ion peak selection, baseline correction, data transfer, peak intensity comparison, ^13^C deisotoping, and quantitation were conducted using a custom programmed Microsoft Excel macro after considering the principles of lipidomics^87^. Given our small sample size and lack of statistical power to resolve putative sex-specific treatment effects, paired analysis of all subjects (males + females) was performed to focus on the effects of senolytic treatment in relation to the participant’s baseline.

### RNA Preparation for Transcriptomic Analyses:

To analyze participant PBMC samples for senolytic induced changes in genes included in the CTRA transcriptomic profile, RNA was isolated from frozen PBMC samples using the QIAGEN protocol for isolation of total RNA from PBMCs outlined with the RNeasy Mini Kit (ca. no. 74104). RNA 260/280, 260/230, and RNA concentration were assessed using NanoDrop, and samples were diluted to 10 ng/μl with RNAse free water. A nanoString nCounter XT CodeSet Gene Expression Panel was designed specifically to measure the 53 gene CTRA transcriptomic profile of these samples. Samples were prepared following the hybridization protocol assay (nCounter XT). CTRA genes were assessed in RNA isolated from PBMC specimens using the nanoString nCounter XT CodeSet Gene Expression Panel, custom designed to specifically measure the 53 genes which make up the CTRA profile. CTRA gene expression levels were normalized to the following housekeeping genes: *HPRT1*, *PGK1*, *POLR2A*, and *TBP*, while *MAPT* was included as negative control for differential gene expression analysis.

### Statistical Analysis:

Baseline to post-treatment changes in plasma and CSF biomarkers were assessed using multiple paired sample t-tests in GraphPad Prism version 9.4.1. Paired t-tests were two-tailed and significance was determined by *P* < 0.05. Lipidomics statistical analysis was performed using the MetaboAnalyst metadata table and paired one factor modules ((https://www.metaboanalyst.ca/). Briefly, lipidomics datasets were transformed (cube root for plasma data expressed relative to protein content and Log_10_ for all other data sets) and scaled (mean centered) so that the data followed a normal distribution. Subclass analyses were performed in GraphPad Prism using multiple paired t-tests. Normalized transcriptomic data were analyzed by moderated t-test implemented in the limma package^88^. Each paired group was treated as a covariate in the design matrix for the paired differentially expressed genes (DEG) analysis between baseline and posttreatment samples. As with the data contained in the original report^8^, *P* values were not corrected for multiple comparisons due to the small sample size and exploratory nature of these reported outcomes. Correlation was assessed with Pearson r analyses with simple linear regression.

## Data Availability

The minimum dataset necessary to interpret, verify, and extend the research presented in this article will be available upon request to the corresponding author. The trial was registered on ClinicalTrials.gov: NCT04063124, the full study protocol^81^ and primary and secondary aims of the study^8^ have been previously published.
